# Dynamics of *Ixodes ricinus* and associated bacterial pathogens in the forest and agricultural ecosystems of northeastern France

**DOI:** 10.1128/aem.00793-25

**Published:** 2025-05-29

**Authors:** Ali Haidar-Ahmad, Cathy Barthel, Rebeca Abigail Rivera-Vallejo, Armand Maul, Marie-Lazarine Poulle, Nathalie Boulanger

**Affiliations:** 1 UR3073: PHAVI: Groupe Borrelia, Institut de Bactériologie, University of Strasbourg 27083, Strasbourg, France; 2 Université de Lorraine, CNRS, LIEC 27035, Metz, France; 3 UR ESCAPE, Université de Reims Champagne-Ardenne (URCA) 27078, Reims, France; 4 CERFE, Université de Reims Champagne-Ardenne (URCA) 27078, Boult-aux-Bois, France; 5 French National Reference Center for Borrelia, Hôpitaux Universitaires, Strasbourg, France; UMR Processus Infectieux en Milieu Insulaire Tropical, Ste. Clotilde, France

**Keywords:** ticks, ecosytem, tick-borne diseases, *Borrelia burgdorferi*

## Abstract

*Ixodes ricinus* ticks evolve mainly in forest ecosystems, which can be affected by human activities in different ways. The present study aimed to analyze the relationship between different landscapes, tick populations, and the circulation of bacterial agents in three French departments, i.e., Ardennes, Marne, and Meuse. A total of eight sites were selected in wetlands, agricultural areas, forest areas, and a hunting park. All these sites host populations of forest rodents, European badger, red fox, and roe deer; some also host wild boar. Ticks were collected during the period of peak activity from March to June over the course of 3 years. *Ixodes ricinus* nymphs were then analyzed by PCR for the presence of the following bacteria: *Borrelia burgdorferi* sensu lato, *Neoehrlichia mikurensis*, *Anaplasma phagocytophilum*, and *Borrelia miyamotoi*. The highest tick density, 145.7 nymphs/100 m^2^ was observed in the hunting park; specifically, in a specific double-fenced enclosure where the vegetation was particularly abundant with large deciduous trees. The presence of various hosts (rodents, badgers, foxes, and roe deer) was recorded. *N. mikurensis* and *B. burgdorferi* s.l., with a prevalence of 11.2% and 9.8%, respectively, were the most detected microorganisms in nymphs. Overall, 25.7% of the tested ticks were positive, and 3.3% carried at least two microorganisms. Forested areas surrounded either by agricultural areas or sites characterized by their host diversity and dense herbaceous vegetation appear suitable for the proliferation of ticks and associated pathogens.

**IMPORTANCE:**

The distribution and abundance of *Ixodes ricinus* ticks and associated pathogens vary according to the ecosystem. Forest areas, characterized by dense vegetation, many large trees, abundant leaf litter, and certain vertebrate hosts, have the highest tick densities. The impact of anthropization on these factors has not been sufficiently studied. In this study, we measured tick abundance in relation to the presence of wild animals (ungulates and rodents) and vegetation data, which are important factors influencing tick proliferation. In addition, we interviewed forestry workers, hunters, and farmers in the study area to identify natural and anthropogenic factors likely to affect tick populations. Overall, we conclude that ecosystem changes, in particular afforestation and deer population expansion, need to be thoroughly assessed in order to develop effective control measures while ensuring climate change mitigation.

## INTRODUCTION

The impact of the anthropization of ecosystems is being increasingly documented as a key element contributing to the increase of tick populations and tick-borne diseases (1). Specifically, landscape fragmentation and connectivity have been identified as important factors (2–5), as well as the expansion of wild ungulates (6, 7), especially roe deer (7, 8).

Variations in climate conditions and socio-ecosystems, which affect forest and wildlife management, are considered to be responsible for the expansion of the *Ixodes ricinus* complex in the temperate zones of the Northern Hemisphere (9–14). This complex is a vector of various potentially pathogenic microorganisms, such as the *Borrelia burgdorferi* sensu lato (s.l.) complex, which is responsible for Lyme disease, but also of re-emerging bacteria such as *Anaplasma phagocytophilum*, *Borrelia miyamotoi* (which is associated with relapsing fever) (15), and *Neoehrlichia mikurensis* (16, 17). *Ixodes ricinus* is also a vector of parasites (e.g., *Babesia microti* and *B. divergens*) (18) and viruses (e.g., tick-borne encephalitis) (15, 19). This tick evolves in specific ecosystems such as mixed forests. Here, it survives in the leaf litter, which provides protection against desiccation, and develops into three stages after egg hatching: larva, nymph, and male and female adults (20, 21).

Northeastern France is known as an endemic area for ticks and tick-borne diseases (22). This area comprises the Alsace region (Bas-Rhin and Haut-Rhin departments) and the Moselle department, which are both densely populated, as well as the Meuse, Marne, and Ardennes departments, which are characterized by rural areas with low human density. In the Alsace region (Bas-Rhin and Haut-Rhin departments), ticks and tick-borne diseases are regularly monitored (23–25). A study conducted in Moselle analyzed the impact of past human activities on ecosystems, soils, and hosts (26), and other studies have revealed the high human seroprevalence of tick-borne diseases in northeastern France (27, 28). However, only a few studies have been conducted on *I. ricinus* and its associated pathogens in the rural areas of the Meuse and Ardennes departments. In these studies, ticks were collected, and some tick-borne pathogens were identified (29, 30); however, neither the ecosystem nor the impact of human activities was thoroughly investigated.

In the present study, we selected different sampling sites in wetlands, forested areas, and a hunting park and collected *I. ricinus* ticks during the period of peak activity, from March to June, for 3 years (2021–2023). We then analyzed the bacterial content of the ticks using molecular methods. By conducting interviews with hunters, forestry workers, and farmers, we identified the main ecosystem-related factors and human activities potentially affecting tick populations and the circulation of pathogens in this region.

## MATERIALS AND METHODS

### Study area, sampling sites, and tick collection

The study was conducted in the Marne, Ardennes, and Meuse departments of northeastern France. These departments are part of the “Zone atelier rurale en Argonne” (ZARG) project and represent 1 of the 16 “workshop areas” designated by the French National Center for Scientific Research. In each of these areas, a network of interdisciplinary studies is conducted, focusing on socio-ecosystems and the environment in relation to societal issues.

Questing ticks were collected during the period of peak activity (from March to June 2021–2023) by dragging a white sponge cloth of 1 m^2^ on vegetation over a transect of 300 m^2^. The cloth was inspected every 10 m, and ticks were collected using tweezers and transferred alive into a tube, which was brought to the laboratory and frozen at −20°C.

Eight sites were sampled in 2021 and 2022 in the three departments: two wetlands (Germont swamp and Belval en Argonne-lake), three forested areas (Dannevoux, Belval en Argonne, and Roban), and three areas within the Belval-Bois-des-Dames fenced hunting park (named La cuve, red deer, and plot 32). Plot 32 was a double-fenced plot for forest regeneration (the sampling sites are described in detail in the Supplementary data).

In 2023, we investigated plot 32 at the Belval-Bois-des-Dames park more thoroughly by adding two additional transects. We also sampled again the two other sites (La cuve and red deer) of Belval park, as well as the Dannevoux forest, as a reference site for the tick ecosystem.

The collected ticks were identified by matrix-assisted laser desorption-ionization time-of-flight mass spectrometry (MALDI-ToF-MS) as described previously (31).

### DNA extraction and detection of bacterial pathogens by PCR

For each site, a maximum of 60 nymphs were individually analyzed. DNA was extracted using ammonium hydroxide (32). For the identification of *B. burgdorferi* s.l., an initial amplification via real-time quantitative PCR (RT-qPCR) targeted the *flagellin* gene and was followed by species-specific RT-qPCR targeting variable regions of this gene for each *Borrelia* species, as described (24). For *Anaplasma phagocytophilum* and *Borrelia miyamotoi*, the *major surface protein 4* gene and the relapsing fever *flagellin* gene were targeted, respectively (33, 34). An RT-qPCR assay adapted from Jahfari et al. (16) targeting the *GroEl* gene was employed for *Neoehrlichia mikurensis*. Positive and negative controls were included in each PCR amplification to ensure the specificity of reactions. All PCR assays were performed on a BioRad Opus 384 system.

### Statistical analyses

The Kruskal–Wallis test was used to compare the density of nymphs (DON) across the eight sites during 4 months in each of the 3 years examined. In case of significant results, the Mann–Whitney–Wilcoxon pairwise post hoc test with Bonferroni correction for multiple comparisons was performed to determine which of the sample pairs were significantly different.

Pearson’s χ test or Fisher’s exact test (when the frequencies allowed calculations) were used to compare (i) the proportions of infected nymphs and (ii) the relative proportions of potentially pathogenic microorganisms by site, month, and year.

It should be noted that the experiment, which was carried out for nymphs counting, was not factorial due to a certain number of missing values (21/73) (Table S1). Such a flaw in the experimental design can lead to confounding effects, in the sense that some effects may not be clearly distinguishable from others. All statistical analyses were performed in R (version 4.2.1).

## RESULTS

We investigated the spatial and temporal variations in the density of nymphs (DON), the nymph infection prevalence (NIP), and the density of infected nymphs (DIN), as well as the distribution of four tick-borne pathogens in infected nymphs.

### Density of nymphs (DON)

Overall, a total of 5152 ticks were collected in the eight sites examined (i.e., three forest areas, two wetland areas, and three different areas within the hunting park) (Table S1). They were all *I. ricinus* nymphs and adults, or *Dermacentor reticulatus* adults. In this manuscript, we only present the data on *Ixodes* nymphs. The density of nymphs/100 m^2^ varied from 2021 to 2023, depending on site, month, and year (Table 1).

**TABLE 1:** Density of nymphs (DON)/100 m^2^ in the different sampling sites from 2021 to 2023^*a*^

Site	March		April			May			June		
	2021	2022	2021	2022	2023	2021	2022	2023	2021	2022	2023
Belval park-La cuve	4.7	0.0	10.3	9.1	10.3	6.7	2.3	10	8.7	10.7	16.3
Belval park-red deer	0.0	0.8	3.0	2.9	4.3	6.7	2.6	4.3	4.7	8.7	13
Belval park-plot 32	52.1	7.7	**145.7^*b*^**	29.4	91.3**	101.9	33.3	80**	93.5	53.3	40.3**
Roban forest	7.6	*	*	4.9	*	*	6.2	*		40.0	*
Belval en Argonne-forest	*	5.0	11.5	6.9	*	9.3	7.3	*	11.3	8.3	*
Dannevoux forest	*	*	24.5	20.5	76.3	*	7.9	70.6	*	22.0	121
Germont swamp	6.3	0.0	8.7	10.8	*	10.3	2.3	*	4.7	3.0	*
Belval en Argonne-lakes	2.3	3.7	4.7	7.4	*	7.3	7.4	*	1.3	4.3	*

^*a*^ *No data; **average of the three collection sites of plot 32 in 2023.

^*b*^ The value in bold represents the highest tick density.

With regard to the spatial variation in DON (Table S2a), statistical analysis showed that there was a “site” effect, i.e., nymph abundances (DON) varied among the eight sites examined (Kruskal–Wallis test, *P* < 10^−4^). The DON values observed in the wetland areas were lower than those in the forest areas, while the DON values recorded in the three sites at the hunting park showed no homogeneity, with Belval park-red deer (mean = 4.6) and Belval park-plot 32 (mean = 66.2) as extremes. Post-hoc pairwise comparisons showed that DON was significantly higher in Belval park-plot 32 than in the other sites (pairwise tests, *P* < 0.03), with the exception of Dannevoux forest (mean = 49.0) and Roban forest (mean = 14.8). DON varied throughout the 4-month periods examined in each year (Kruskal–Wallis test, *P* = 0.011), and post-hoc pairwise comparisons revealed that it was lowest in March (*P* < 0.06) (Table S2b). DON also varied across the years examined (Kruskal–Wallis test, *P* = 0.011), with the values in 2023 (mean = 44.8) being significantly higher than those in 2021 (mean = 21.9) and 2022 (mean = 11.0) (pairwise tests, *P* < 0.01) (Table S2c).

### Nymphal infection prevalence (NIP)

**Fig 1:**
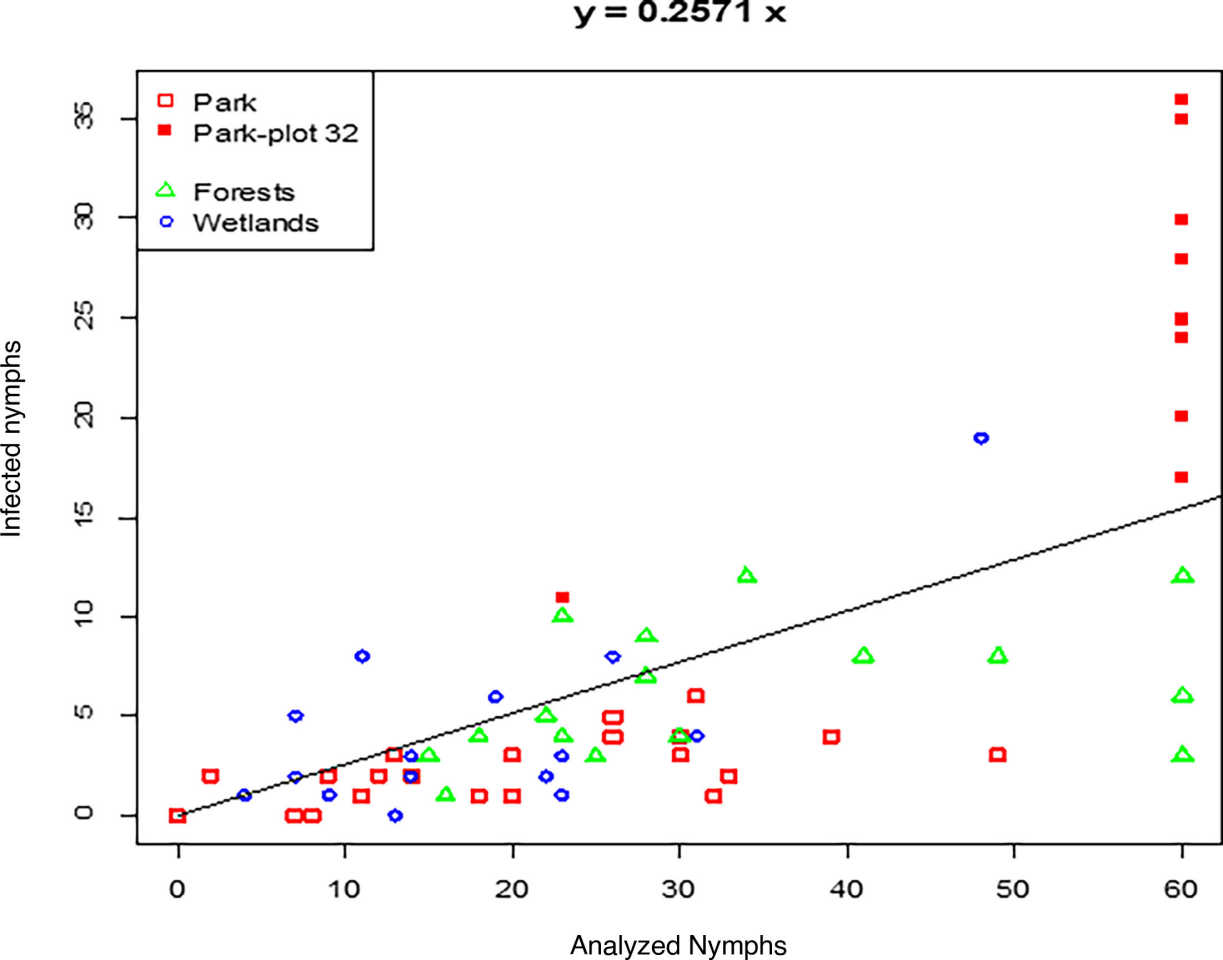
Infected nymphs versus analyzed nymphs within the three ecosystems examined. The filled red squares represent the samples collected at Belval park-plot 32.

The nymphs were analyzed for the presence of tick-borne pathogens. An increasingly monotonic relationship was observed between the number of infected nymphs and nymph abundance (Kendall’s τ = 0.593, *P* < 10^−4^), as shown by the scatter plot in Fig. 1. The analysis showed that 25.7% (95% CI 23.8%–27.7%) of the nymphs were infected in all the sampling sites. However, it should be noted that the actual proportion of infected nymphs may be slightly lower due to co-occurrences, which were estimated to occur in 3.3% (95% CI, 2.6–4.2%, 65/1976) of all tested ticks.

NIP values differed significantly among the eight sites examined (χ test, *P* < 10^−4^) (Table S3a). Values were lower at Belval fenced park-La cuve (9.1%; 95% CI 6.2%–13.0%, 25/276), Dannevoux forest (15.0%; 95% CI 11.7%–19.1%, 53/353), and Belval park-red deer (15.6%; 95% CI 10.7%–22.1%, 24/154) sites, and higher at Belval park-plot 32 (45.1%; 95% CI 41.2%–49.0%, 281/623).

The proportion of infected nymphs varied throughout the 4-month periods examined in each year (Fisher’s exact test, *P* < 10^−4^) (Table S3b). NIP values were lowest in June (22.8%; 95% CI 19.7%–26.2%, 147/645) and May (23.0%; 95% CI 19.6%–26.8%, 119/517) and highest in March (37.7%; 95% CI 31.0–44.9%, 69/183).

No significant variation in NIP was detected among the 3 years examined (Fisher’s exact test, *P* = 0.567) (Table S3c). Specifically, the observed values were 25.3% (95% CI 22.2%–28.7%, 172/679), 24.9% (95% CI 21.9%–28.1%, 190/764), and 27.4% (95% CI 23.8%–31.3%, 146/533) for 2021, 2022, and 2023, respectively, which are consistent with the estimated proportion for the study as a whole, i.e., 25.7% (95% CI 23.8–27.7%).

**Fig 2:**
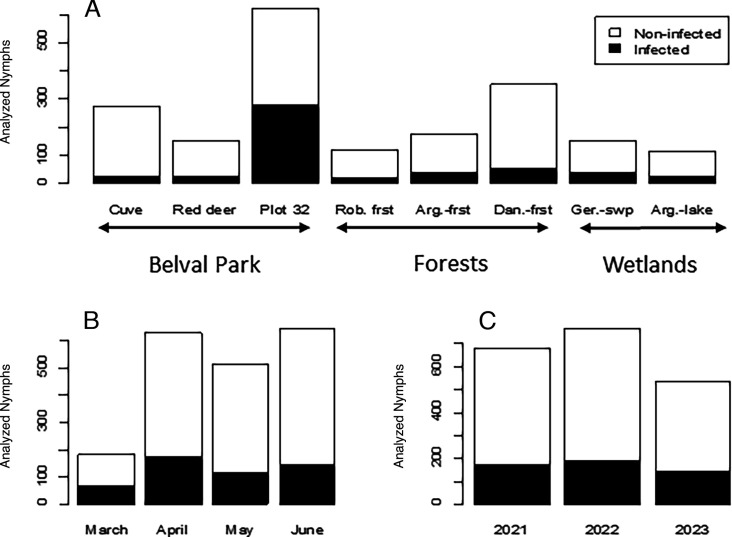
(**A**) Numbers of infected and non-infected nymphs at different sites within the three main ecosystems examined: Belval fenced park (La cuve, red deer, and plot 32), forest areas, and wetland areas. Abbreviations: Rob, Roban; Arg, Belval en Argonne; Dan, Dannevoux; Ger, Germont; Frst, Forest; and Swp, Swamp. Portions B and C indicate the numbers of infected and non-infected nymphs across months and years, respectively.

The proportions of infected nymphs are illustrated in Fig. 2.

### Analysis of DIN and associated microorganisms

The prevalence of the four potentially pathogenic microorganisms detected in this study, i.e., *B. burgdorferi* s.l. (BOR), *A. phagocytophilum* (ANA), *B. miyamotoi* (BMY), and *N. mikurensis* (NEH) is shown in Table 2.

**TABLE 2:** Bacterial prevalence in *Ixodes ricinus* nymphs^*a*^

Parameter	Value for:				
	BOR	ANA	BMY	NEH	Whole
No. positive/1,976 analyzed ticks	193	30	63	222	508
% positive	9.8	1.5	3.2	11.2	25.7
95% CI	8.5–11.2	1.1–2.2	2.5–4.1	9.9–12.7	23.8–27.7

^*a*^ BOR, *Borrelia burgdorferi sensu lato*; ANA, *Anaplasma phagocytophilum*; BMY, *Borrelia miyamotoi*; and NEH, *Neoehrlichia mikurensis*.

**Fig 3:**
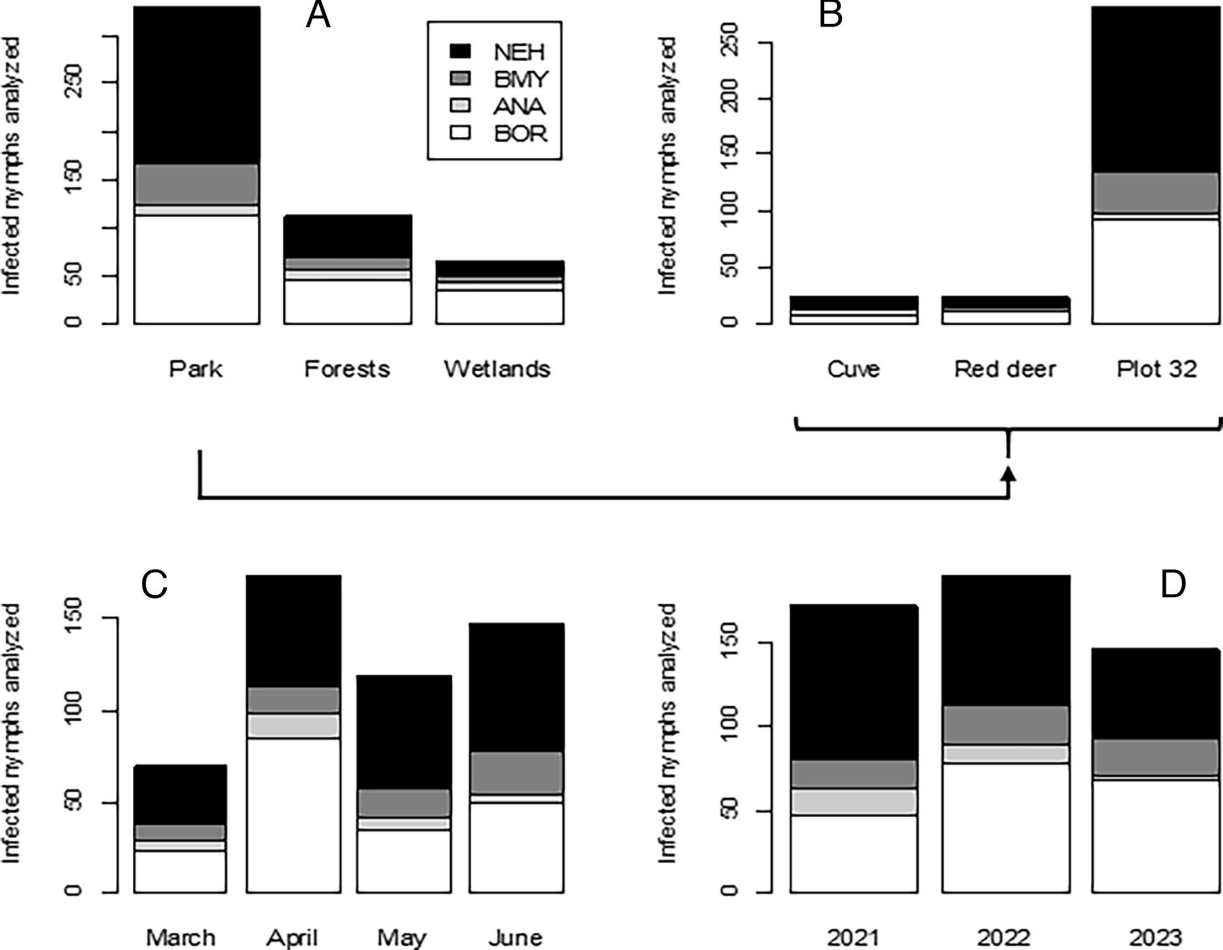
Relative proportions of the four potentially pathogenic bacteria in infected nymphs within the three ecosystems examined (**A**) and in the plots inside the fenced park (**B**) as well as across months (**C**) and years (**D**). Abbreviations: BOR, *Borrelia burgdorferi sensu lato*; ANA, *Anaplasma phagocytophilum*; BMY, *Borrelia miyamotoi*; and NEH, *Neoehrlichia mikurensis*.

The variation in the relative proportions of these four bacteria was examined across sites, months, and years. Their abundances are presented in Table S4a through d, respectively. The corresponding proportions (prevalence) of these microorganisms in infected nymphs are shown in Fig. 3.

As with all infections, the proportions of the four pathogenic bacteria varied among sites (χ test, *P* < 10^−3^) and across months (*P* < 0.01) and years (*P* < 10^−3^).

The distribution of the four bacteria varied among the three ecosystems examined (fenced park, forests, and wetlands). More specifically, the variations between the relative prevalence of the four bacteria and the type of environment concern the park and wetlands, while their prevalence observed for forests was close to the average.

In Belval park, the proportion of *A. phagocytophilum* (ANA) among infected nymphs was lower (3.6%; 95% CI 2.1%–6.2%, 12/330) than the average (5.9%), whereas that of *N. mikurensis* (NEH) was higher (49.7%; 95% CI 44.3%–55.1%, 164/330) than the average (43.7%). This high proportion of NEH was especially observed in plot 32 (52.3%; 95% CI 46.5–58.1%, 147/281).

The opposite was observed in the wetland areas, where the corresponding proportions for ANA and NEH were 13.6% (95% CI 7.3%–23.9%, 9/66) and 24.2% (95% CI 15.5%–35.8%, 16/66), respectively. In the wetlands, *B. burgdorferi* s.l. (BOR) (51.5%; 95% CI 39.7%–63.1%, 34/66) was also above average (38.0%). The proportion of *Borrelia miyamotoi* (BMY) was stable in all three ecosystems (around 12.4%, Table S4b).

Statistical analysis of monthly variations showed that the relative prevalence of BOR decreased from April (49.1%; 95% CI 41.8%–56.5%, 85/173) to May (29.4%; 95% CI 22.0%–38.1%, 35/119), while the opposite was true for NEH, whose prevalence increased from 34.7% (95% CI 28.0%–42.0%, 60/173) in April to 52.1% (95% CI 43.2–60.9%, 62/119) in May (Table S4c).

Throughout the 3-year period, the prevalence of BOR increased from 27.3% (95% CI 21.2%–34.4%, 47/172) in 2021 to 46.6% (95% CI 38.7%–54.6%, 68/146) in 2023, while that of ANA (NEH) decreased from 9.3% (95% CI 5.8%–14.6%, 16/172) to 2.1% (95% CI 0.7%–5.9%, 3/146) (resp. 53.5%; 95% CI 46.0%–60.8%, 92/172 to 36.3%; 95% CI 28.9%–44.4%, 53/146) over the same period (Table S4d).

As for BMY, its relative prevalence can be considered stable at around 12.4% (95% CI 9.8–15.5%, 63/508) over the 3-year period.

**Fig 4:**
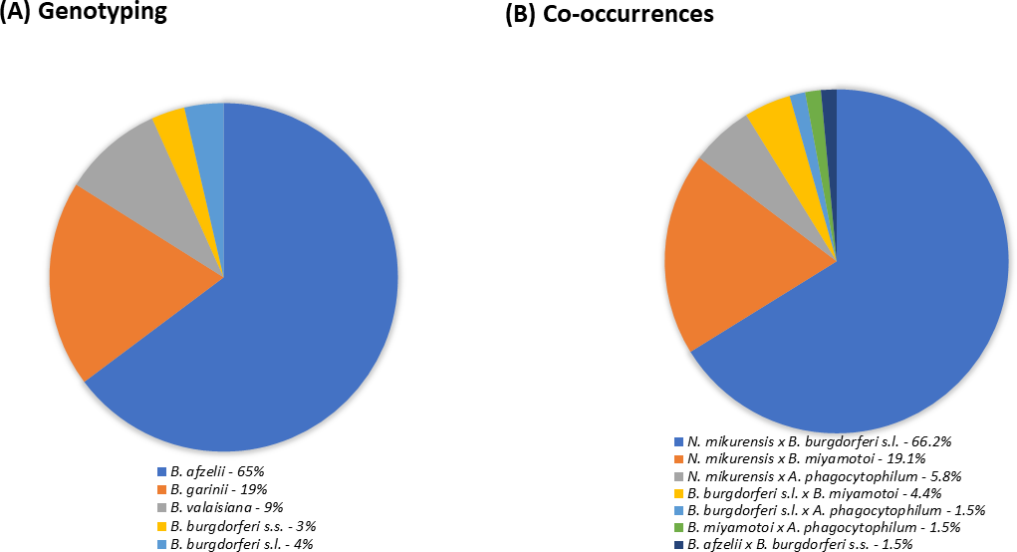
(**A**) Abundances of *Borrelia* spp. throughout the 3-year period, as revealed via genotyping. The prevalence of infection with *B. burgdorferi* sl was 9.8%. Global prevalence of infected ticks: 25.7%. (**B**) Globally, co-occurrence with different bacteria was observed in 12.8% of infected ticks and 3.3% of all tested ticks.

The *Borrelia* spp. were subsequently genotyped, and the most abundant species were identified as *B. afzelii* (65%), *B. garinii* (19%), *B. valaisiana* (9%), *B. burgdorferi* s.s. (3%), and *B. burgdorferi* s.l. (4%) (Fig. 4A). A certain degree of tick co-occurrence was also detected. The proportions were ranked as follows: 66.2% *N*. *mikurensis*/*B. burgdorferi* s.l. (the most abundant), 19.1% *N*. *mikurensis/B. miyamotoi*, 5.8% *N*. *mikurensis/A. phagocytophilum*, 4.4% *B. burgdorferi sl/B. miyamotoi*, 1.5% *B. burgdorferi sl/A. phagocytophilum*, and 1.5% *B. miyamotoi/A. phagocytophilum* (Fig. 4B). Finally, 12.8% (65/508) of the infected ticks and 3.3% (65/1976) of all the tested ticks were co-infected.

Overall, the two most abundant bacteria were *B. burgdorferi* s.l. and *N. mikurensis*. With regard to DIN, the highest value was recorded in April 2023 at plot 32 in Belval park, with 53.3 infected nymphs/100 m^2^ (Table 3). At this time, the DIN value at the reference forest site (Dannevoux) was also high compared with that at the other sites.

**TABLE 3:** Density of infected nymphs/100 m^2^ (DIN)^*a*^

Site	March		April			May			June		
	2021	2022	2021	2022	2023	2021	2022	2023	2021	2022	2023
Belval park-la Cuve	0.3	0	0.6	1.8	1.0	0.3	0	1.3	1.3	0.33	1.0
Belval park–red deer	0	0.8	0.7	0.3	1.0	1.0	0	0.7	0.7	1.7	1.3
Belval Park-Plot 32	20.8	3.7	48.6	13.7	**53.3^*b*^**	28.9	11.1	48.0	39.0	26.7	23.5
Roban-forest	2.4	*	*	1.1	*	*	0.4	*	*	4.0	*
Belval/Argonne - forest	*	1.0	2.0	1.6	*	2.3	1.0	*	4.0	1.0	*
Dannevoux forest	*	*	4.0	4.0	15.3	*	3.4	3.5	*	2.2	12.1
Germont swamp	2.0	0	2.7	4.3	*	1.3	0.7	*	0.7	0.3	*
Belval/Argonne - lakes	1.6	2.7	1.0	0.3	*	0.7	1.0	*	0.3	0	*

^*a*^ *No data.

^*b*^ The value in bold represents the highest density of infected nymphs.

## DISCUSSION

This preliminary study investigated the influence of various ecosystems on the presence of ticks and associated tick-borne bacteria in three departments of northeastern France included in the ZARG project. Ticks were collected from three different ecosystems, i.e., wetlands (two sites), forested areas (three sites), and a 1,000 ha fenced hunting park (three sites). The two wetlands are nature reserves.

The ecosystem type seemed to play a significant role in the *I. ricinus* density and associated pathogens (Table S5). Wetlands as well as the red deer site at Belval Park, where a red deer population (*Cervus elaphus*) is present, exhibited the lowest tick densities. At Belval Park, deer have heavily trampled the soil and removed a lot of the vegetation, which has rendered the environment inhospitable for ticks. As nature reserves, the two wetlands are maintained through regular mowing along the water’s edge, which creates open spaces with abundant sunlight. As expected, the three forested sites presented a significant density of ticks because the conditions in this habitat are the most conducive for their proliferation. Notably, the plot 32 transect in Belval Fenced Park had 10 times as many ticks as most of the other sites. This plot had a high vegetation density, with abundant grass and leaf litter and numerous brambles and large trees.

The presence of different hosts is essential for tick survival, especially in the case of a generalist tick like *I. ricinus* (21). Wild ungulates, especially roe deer, constitute essential hosts for adult ticks during reproduction, but they are not affected by *Borrelia* (8, 35, 36). When roe deer populations are not contained, tick populations expand. Wild animals were abundant in all the investigated sites, especially in plot 32 at Belval Hunting Park (Table S6). Here, we observed deer, rodents, foxes, and badgers. An important wild ungulate population is distributed in Eastern France (Fig. S1). In 2023, the “Office Français de la Biodiversité” recorded the hunting of 7376 (Ardennes), 8878 (Marne), and 10296 (Meuse) roe deer (37). These numbers are particularly high for France. However, rodents and birds are important hosts for tick larvae and nymphs. It would also be important to monitor rodent and bird abundance in specific areas of interest to measure their impact on tick abundance and tick-borne bacteria prevalence.

Among the four tested pathogens, NEH was the most detected in *Ixodes* nymphs, especially in Belval-plot 32, followed by BOR. NEH was first detected in *I. ricinus* in the Netherlands in specific ecosystems (38) and was later identified in Germany as a potential human pathogen (39). Interestingly, in France, this bacterium is frequently detected in *I. ricinus* in the eastern part of the country but is rarely found in the western part (40), which highlights the roles of the ecosystem and host circulation. The four pathogens were present in all ecosystems during the peak of tick activity in April, but no significant variation in DIN was observed from one year to the next. Interestingly, in the plot 32 transect, all four pathogens were highly abundant, which suggested the presence of abundant rodent communities (*Myodes*, *Apodemus*, or *Microtus* spp.) serving as reservoirs (41, 42). Indeed, rodents serve this purpose for *N. mikurensis* and *B. afzelii*. For BOR, *B. afzelii* was the most abundant genospecies, followed by *B. garinii* and *B. valaisiana*, which are mainly associated with birds (42).

A previous study of *I. ricinus* female adults conducted in 2012 in the Ardennes screened a total of 38 microorganisms via high-throughput real-time PCR and revealed that 45% of the ticks were infected by at least one microorganism (43). In the present study, by testing the nymph stages and only four pathogens known to be potentially pathogenic to humans, we found that 25.7% of ticks harbored at least one of them. The proportion of ticks carrying Lyme spirochetes detected (40) was 21.7%, while that observed in our study for the three departments (Ardennes, Meuse, and Marne) was 9.8%. This is not surprising, as the nymphal ticks would have had one less (potentially infected) blood meal than the adult female ticks. Compared to the previous study (40), we found lower proportions of *B. afzelii* (6.3% versus 9.4%), *B. garinii* (1.9% versus 10.8%), and *B. valaisiana* (0.9% versus 9%). The proportions of *A. phagocytophilum* and *B. miyamotoi* were also lower, while that of *N. mikurensis* was considerably higher (38).

Among the detected co-occurrences, the association between *N. mikurensis* with *B. burgdorferi* s.l. was the most abundant of the different co-occurrences (66.2%). We noticed a very frequent association between *N. mikurensis* and other potentially pathogenic microorganisms as well; a phenomenon that has already been observed in other places in Europe (44). For example, in different forested areas in the Netherlands, the most prevalent association was shown to be that between *N. mikurensis* and *B. afzelii* (9). Rodents (*Myodes*, *Apodemus*, or *Microtus* spp.) are suspected to be the main hosts allowing this association. In this same study and others, roe deer were considered to be responsible for the association between *A. phagocytophilum* and *N. mikurensis* (45). It should be noted that, while relatively frequent in ticks, co-occurrences are rare in humans (46). In the present study, the prevalence of *B. miyamotoi* and *A. phagocytophilum* in ticks was 3.2% and 1.5%, respectively, which corresponds to the range reported in previous studies conducted in the Alsace (23–25) and Meuse (29) regions.

Vegetation is very important for *Ixodes* tick populations, as it provides sufficient relative humidity for their survival and can thus represent a determinant factor for either the dilution or amplification of tick habitats (1, 47). A forest with abundant vegetation and leaf litter facilitates the survival of *Ixodes* ticks, which spend their life climbing on vegetation in search of a vertebrate host (48, 49). In eastern France, a significant proportion of the territory is covered by forests: 32% in Ardennes, 37% in Meuse, and 20% in Marne (50). After World War II, which greatly impacted this region, the French government promoted extensive afforestation programs (artificial tree planting). As a result, between 1946 and 1987, 2.3 million hectares were reforested in mainland France. Over the course of 53 years, the French woodland area increased by a third, from 10.7 million hectares in 1946 to 15.2 million hectares in 1999 (51). Tick expansion associated with reforestation after the abandonment of agricultural fields has similarly been observed in Europe (1) and in the USA, but for different reasons (11). Since 1945, major environmental changes have occurred in France, and according to the testimonies of farmers, ticks are now quite abundant. Small farms with pasture, hedgerows, and orchards have disappeared and have been replaced by large farms for intensive corn and wheat cultivation. The impact of woodland expansion on ticks has also been examined in Scotland, where mature woodlands were found to be suitable environments for ticks (35). This has been observed globally in Europe (52). As a matter of fact, eastern France is also one of the two main regions where Lyme borreliosis is endemic, along with central France (53).

In a previous study, we mentioned the risk of fragmented forests, like in agricultural areas, where forest fragmentation provides refuge for animals. In this specific ecosystem, we found 10 times as many *I. ricinus* ticks as in a large forested area, as well as a higher prevalence of pathogens (26). In this sense, the forested area in plot 32 examined in the present study constitutes another very interesting example of an ecosystem particularly suitable for ticks and tick-borne pathogens. This enclosure for forest regeneration, with its large trees, important coppice area, shrub cover, and abundance of brambles, as well as its different animal hosts, showed the highest pathogen prevalence. Interestingly, between May and June 2023, after fences were removed in this enclosure, we were able to collect considerably fewer ticks, at least in two out of the three transects (Table S1).

As mentioned in Fish (3, 11), “the history of forests is tightly linked to the history of deer populations.” The expansion of Lyme borreliosis and other tick-borne diseases should make us reflect on the optimal levels of animal and plant biodiversity required in order to limit the impact of climate change and, at the same time, control the spread of zoonoses associated with ticks (1). Within the context of climate change, it is important to thoroughly assess the impact of increased biodiversity and vegetation on ticks and tick-borne diseases in order to develop control strategies.

## SUPPLEMENTAL MATERIAL

Supplemental material aem.00793-25-s0001.docx Site description, Tables S1 to S6, and Fig. S1.

ASM does not own the copyrights to Supplemental Material that may be linked to, or accessed through, an article. The authors have granted ASM a non-exclusive, world-wide license to publish the Supplemental Material files. Please contact the corresponding author directly for reuse.
