# Supplementary material for: Dynamics of Ixodes ricinus and associated bacterial pathogens in the forest and agricultural ecosystems of northeastern France
Source: Appl Environ Microbiol. 2025 May 29;91(6):e00793-25. doi: 10.1128/aem.00793-25 (PMC12175513; doi:10.1128/aem.00793-25)
Supplement: Supplemental material — Site description, Tables S1 to S6, and Fig. S1. [file aem.00793-25-s0001.docx]

**
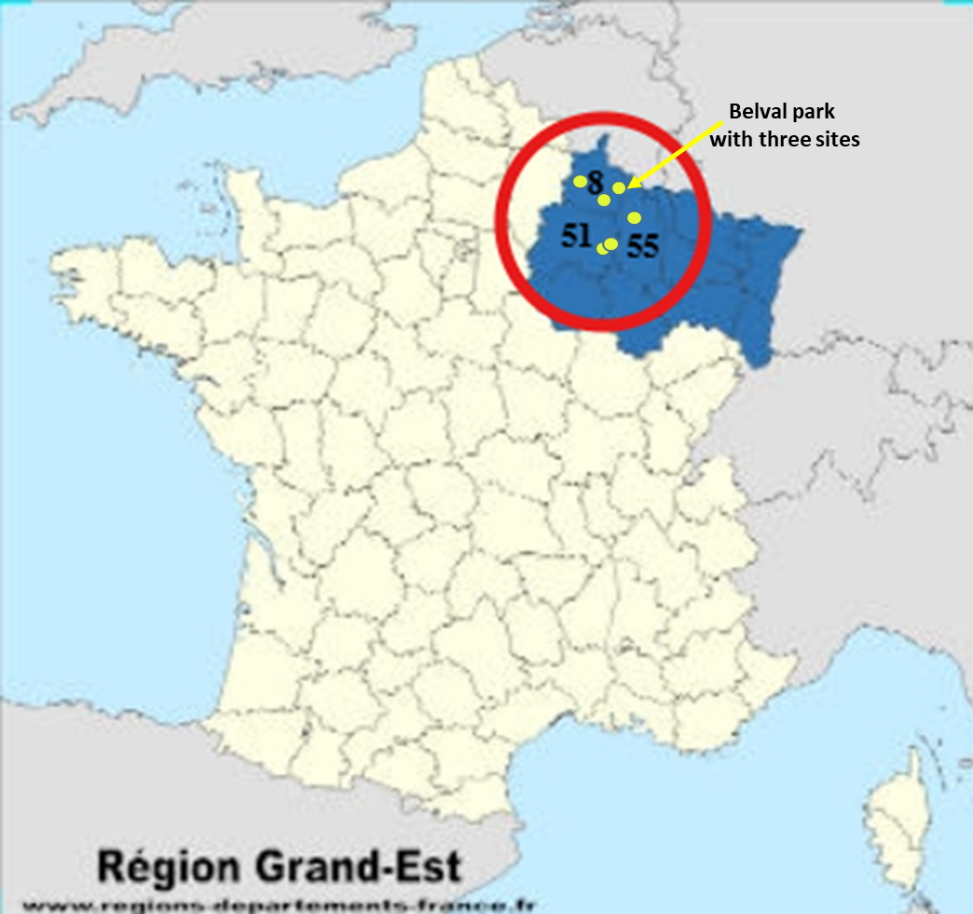
Supplementary data**

**Site description :**

1. **Belval fenced park**: **Sites 1 to 3:** is located in the **Ardennes department (8),** in the commune of Belval Bois des Dames, in the “Crêtes Ardennaises” natural region. It is a former hunting park taken over by the François Sommer Foundation. The property was mainly dedicated to large game, with high densities that have varied over time, but which have virtually prevented any renewal of the forest. This means that the forest stands are mainly made up of large and very large trees, with small and medium-sized trees in short supply. Over the last ten years, large-animal densities have hovered around 12 red deer (*Cervus elaphus*) per 100 ha, 40-50 wild boar (*Sus* *scrofa*) per 100 ha and a small roe deer (*Capreolus capreolus*) population that manages to hold its own despite the competition for food and space from red deer and wild boar. The 1,050-hectare enclosed estate comprises 960 ha of deciduous forest, 40 ha of permanent grassland and 50 ha of ponds (4 lakes) and marshes. Pedunculate oak ((*Quercus robur*) is the main species (65%), mixed with beech ((*Fagus* sp., 10%), ash (*Carpinus* sp., 10%), sycamore maple (*Acer pseudoplatanus*, 10%) and 5% various precious trees (wild cherry, elm, lime, etc.). Elevations range from 174 to 291 meters.

In the Belval fenced park, 3 collection plots were selected: “La cuve”, “Red deer” and “plot 32”. The latter was originally an enclosed forest regeneration paddock. A small roe deer population of with an enclosure. Roe deer were kept in this enclosure. Rodent fauna is also abundant. The ground is heavily vegetated with brambles. The ground in the “Red deer” plot is sparsely vegetated, as it is trampled by the animals. The "La cuve" plot forest is an old-growth deciduous forest with a small population of roe deer and wild boar

1. **Forested areas**

**Site 4:** Dannevoux forest. This is a small forested area of 200Ha in an agricultural zone in **the Meuse department (55).** It is mainly made up of deciduous trees and is very marked by the Great War. Few wild boars but many roe deer are present. This is the reference forest.

**Site 5:** Roban forest. This is an old mixed-wood forest. Located in the Ardennes (8).

**Site 6:** Belval en Argonne-forest. Located in the Marne department (51), this is an old-growth forest of mainly deciduous trees.

1. **Wetlands :**

**Site 7:** Germont swamp. Classified as a Natura 2000 site, the Germont marsh is located **in the Ardennes (8),** in an area where cattle farming and arable farming coexist. It is a typical alkaline peatland habitat in the process of being afforested, with environments of running and stagnant water. It covers an area of 100 hectares.

**Site 8:** Belval en Argonne-lakes are located **in the Marne department (51).** They are part of the “Conservatoire des espaces naturels Champagne-Ardennes”. They have been classified as a "Regional Nature Reserve" since 2012.

**Table S1 :** Number of *Ixodes ricinus* nymphs collected on all sites from 2021 to 2023.

In brown: Belval park, in green: the different forests and in blue: swamp or lake.

|  | **Number of *Ixodes ricinus* collected nymphs** | | | | | | | | | | |  | |
| --- | --- | --- | --- | --- | --- | --- | --- | --- | --- | --- | --- | --- | --- |
| **Type de site** | **March** | | **April** | | | **May** | | | **June** | | |  | |
|  | **2021** | **2022** | **2021** | **2022** | **2023** | **2021** | **2022** | **2023** | **2021** | **2022** | **2023** | **Total** |  |
| **Belval Park - La cuve** | 18 | 0 | 33 | 31 | 30 | 20 | 7 | 30 | 26 | 32 | 49 | **276** |  |
| **Belval Park– Red deer** | 0 | 2 | 9 | 11 | 13 | 20 | 8 | 12 | 14 | 26 | 39 | **154** |  |
| **Belval Park– plot 32**   - **Transect 1** - **Transect 2** - **Transect 3** | 172 | 23 | 306 | 91 | 284  345  192 | 214 | 100 | 256  276  188 | 187 | 112 | 242  53  68 | **1987**  **674**  **448** |  |
| **Roban - forest** | 28 | * | * | 18 | * | * | 16 | * | * | 120 | * | **182** |  |
| **Belval en Argonne - forest** | * | 15 | 23 | 22 | * | 28 | 30 | * | 34 | 25 | * | **177** |  |
| **Dannevoux forest** | * | * | 49 | 41 | 229 | * | 23 | 212 | * | 66 | 363 | **983** |  |
| **Germont - swamp** | 19 | 0 | 26 | **48**** | * | 31 | 7 | * | 14 | 9 | * | **154** |  |
| **Belval en Argonne - lakes** | 7 | 11 | 14 | 23 | * | 22 | 23 | * | 4 | 13 | * | **117** |  |
| **Total** | 244 | 51 | 460 | **285** | 1093 | 335 | 214 | 974 | 279 | 403 | 814 | **5152** |  |

* Missing data; counting not carried out; **: adult ticks have been tested.

**Tables S2: Site, month and year relative to mean and median** **DON (100m^2^)**

| Mean  Median | Belval Park –red deer | Belval/Argonne-Lake | Germont swamp | Park Belval-La cuve | Belval/Argonne-forest | Roban forest | Dannevoux forest | Belval park -plot 32 |
| --- | --- | --- | --- | --- | --- | --- | --- | --- |
| Mean | 4.6 | 4.8 | 5.8 | 8.2 | 8.5 | 14.8 | 49.0 | 66.2 |
| Median | 4.3 | 4.5 | 5.5 | 9.1 | 8.3 | 6.9 | 24.5 | 53.3 |

Table S2a: **Site** ranking relative to mean DON (100m^2^)

|  | March | May | April | June |
| --- | --- | --- | --- | --- |
| Mean | 7.2 | 20.9 | 25.4 | 25.8 |
| Median | 4.2 | 7.4 | 10.3 | 11 |

Table S2b: **Month** ranking relative to mean or median DON (100m^2^)

|  | 2022 | 2021 | 2023 |
| --- | --- | --- | --- |
| Mean | 11.0 | 21.9 | 44.8 |
| Median | 7.4 | 7.6 | 28.3 |

Table S2c: **Year** ranking relative to mean or median DON (100m^2^)

**Tables S3: Nymph infection prevalence (NIP)**

|  | Belval Park –La cuve | Belval Park -red deer | Belval Park -plot 32 | Roban Forest | Belval/  Argonne-forest | Dannevoux forest | Germont swamp | Belval/  Argonne-lake | Total |
| --- | --- | --- | --- | --- | --- | --- | --- | --- | --- |
| Infected  nymphs | 25 | 24 | 281 | 21 | 38 | 53 | 42 | 24 | 508 |
| Non-infected | 251 | 130 | 342 | 101 | 139 | 300 | 112 | 93 | 1468 |
| Total | 276 | 154 | 623 | 122 | 177 | 353 | 154 | 117 | 1976 |

Table S3a: Nymph infection prevalence (NIP) for the 8 sites

|  | March | April | May | June | Total |
| --- | --- | --- | --- | --- | --- |
| Infected nymphs | 69 | 173 | 119 | 147 | 508 |
| Non-infected | 114 | 458 | 398 | 498 | 1468 |
| Total | 183 | 631 | 517 | 645 | 1976 |

Table S3b: Nymph infection prevalence (NIP) for the 4 months

|  | 2021 | 2022 | 2023 | Total |
| --- | --- | --- | --- | --- |
| Infected nymphs | 172 | 190 | 146 | 508 |
| Non-infected | 507 | 574 | 387 | 1468 |
| Total | 679 | 764 | 533 | 1976 |

Table S3c: Nymph infection prevalence (NIP) for the 3 years

**Tables S4: the bacterial pathogens**

Tested bacteria: BOR: *Borrelia burgdorferi* sensu lato, ANA: *Anaplasma phagocytophilum*, BMY: *Borrelia myiamotoi*, NEH: *Neoehrlichia mikurensis*

|  | BOR | ANA | BMY | NEH | Total |
| --- | --- | --- | --- | --- | --- |
| Belval park – La cuve | 9 | 4 | 3 | 9 | 25 |
| Belval park - red deer | 11 | 1 | 4 | 8 | 24 |
| Belval park - plot 32 | 92 | 7 | 35 | 147 | 281 |
| Roban forest | 10 | 0 | 2 | 9 | 21 |
| Belval/Argonne-forest | 15 | 4 | 6 | 13 | 38 |
| Dannevoux forest | 22 | 5 | 6 | 20 | 53 |
| Germont swamp | 28 | 5 | 3 | 6 | 42 |
| Belval/Argonne-lake | 6 | 4 | 4 | 10 | 24 |
| Total | 193 | 30 | 63 | 222 | 508 |

Table S4a: **Pathogen** abundance for each bacterium *versus* sites

|  | BOR | ANA | BMY | NEH | Total |
| --- | --- | --- | --- | --- | --- |
| Park | 112 | 12 | 42 | 164 | 330 |
| Forests | 47 | 9 | 14 | 42 | 112 |
| Wetlands | 34 | 9 | 7 | 16 | 66 |
| Total | 193 | 30 | 63 | 222 | 508 |

Table S4b: **Bacteria** abundance *versus* sites (after grouping of sites)

|  | BOR | ANA | BMY | NEH | Total |
| --- | --- | --- | --- | --- | --- |
| March | 23 | 6 | 9 | 31 | 69 |
| April | 85 | 13 | 15 | 60 | 173 |
| May | 35 | 7 | 15 | 62 | 119 |
| June | 50 | 4 | 24 | 69 | 147 |
| Total | 193 | 30 | 63 | 222 | 508 |

Table S4c: Bacteria abundance *versus* months

|  | BOR | ANA | BMY | NEH | Total |
| --- | --- | --- | --- | --- | --- |
| 2021 | 47 | 16 | 17 | 92 | 172 |
| 2022 | 78 | 11 | 24 | 77 | 190 |
| 2023 | 68 | 3 | 22 | 53 | 146 |
| Total | 193 | 30 | 63 | 222 | 508 |

Table S4d: Bacteria abundance *versus* years

**Table S5:** Main vegetation of the different sites.

In brown: Belval Park; in green: forests and in blue: swamp or lake.

| Vegetation | **Dannevoux**  **forest**  **(55)** (49.3074, 5.23610) | **Roban forest**  **(08)**  (49.718593, 4.379166) | **Belval Park**  **(08)**  (49.491537, 5.032495) | | | **Germont**  **Swamp**  **(08)** (49.438131 4.894778) | **Natural reservation**  **Belval en Argonne**  **(51)**  (48.959942, 4.990875) | |
| --- | --- | --- | --- | --- | --- | --- | --- | --- |
|  |  |  | « La Cuve » | « Red deer » | « Plot 32 » |  | Lake | Forest |
| Leaf litter | ++ | + | ++ | + | +++ | Absent | + | ++ |
| Tall grass | Absent | + | + | +++ | +++ | + | ++ | Absent |
| Mowing grass | Absent | Absent | Absent | Absent | Absent | +++ | Absent | Absent |
| Bramble | + | + | + | + | +++ | Absent | Absent | + |
| Pedunculate oak | +++ | +++ | +++ | +++ | +++ | Absent | Absent | ++ |
| Beech, ash, maple | +++ | +++ | + | + | + |  | ++ | ++ |
| Ash willow (*Salix cinerea*) | Absent | Absent | Absent | Absent | - | +++ | + | Absent |

**Table S6** : Main Wildlife of the 8 different sites according hunting federations.

brown: Belval Park ; green: forests and blue: swamp or lake.

| **Wildlife** | Dannevoux Forest (55) (49.3074, 5.23610) | **Ro**ban forest (08) (49.718593, 4.379166) | Park Belval Bois des Dames (08)  (49.491537, 5.032495) | | | Germont swamp  (08) (49.438131 4.894778) | Natural reservation, of Belval en Argonne (51)  (48.959942, 4.990875) | |
| --- | --- | --- | --- | --- | --- | --- | --- | --- |
|  |  |  | « La Cuve » | « Red deer » | « Plot 32 » |  | Lakes | Forest |
| Roe deer (*Capreolus capreolus*) | +++ | +++ | ++ | + | +++ | + | +++ | +++ |
| Red deer (*Cervus elaphus*) | Absent | + | Absent | +++ | Absent | Absent | + | + |
| Wild boar (*Sus scrofa*) | +++ | +++ | + | +++ | Absent | ++ | ++ | +++ |
| Raccoon (*Procyon*) | + | Absent | Absent | Absent | Absent | Absent | Absent | Absent |
| Muskrat (*Ondatra*) | Absent | Absent | Absent | + | Absent | +++ | ++ | Absent |
| Coypu | Absent | Absent | Absent | Absent | Absent | +++ | ++ | Absent |
| Mulots  (*Apodemus* sp.) | +++ | +++ | +++ | +++ | +++ | +++ | +++ | +++ |
| Musaraignes (*Sorex, Crocidura*) | +++ | +++ | +++ | +++ | +++ | +++ | +++ | +++ |
| Forest avifauna | +++ | +++ | +++ | ++ | +++ | + | ++ | +++ |
| Aquatic avifauna | Absent | Absent | Absent | Absent | Absent | +++ | +++ | Absent |
|  |  |  |  |  |  |  |  |  |

**Figure S1 : Data from Office Française de La Biodiversité - OFB (**[Les espèces chassables](https://www.ofb.gouv.fr/les-especes-chassables#tableaux_ongules))
